# State-of-the-Art Ceramic Membranes for Oily Wastewater Treatment: Modification and Application

**DOI:** 10.3390/membranes11110888

**Published:** 2021-11-19

**Authors:** Mingliang Chen, Sebastiaan G. J. Heijman, Luuk C. Rietveld

**Affiliations:** Sanitary Engineering, Department of Water Management, Faculty of Civil Engineering and Geosciences, Delft University of Technology, Stevinweg 1, 2628 CN Delft, The Netherlands; S.G.J.Heijman@tudelft.nl (S.G.J.H.); L.C.Rietveld@tudelft.nl (L.C.R.)

**Keywords:** ceramic membrane, membrane fouling, membrane modification, oily wastewater

## Abstract

Membrane filtration is considered to be one of the most promising methods for oily wastewater treatment. Because of their hydrophilic surface, ceramic membranes show less fouling compared with their polymeric counterparts. Membrane fouling, however, is an inevitable phenomenon in the filtration process, leading to higher energy consumption and a shorter lifetime of the membrane. It is therefore important to improve the fouling resistance of the ceramic membranes in oily wastewater treatment. In this review, we first focus on the various methods used for ceramic membrane modification, aiming for application in oily wastewater. Then, the performance of the modified ceramic membranes is discussed and compared. We found that, besides the traditional sol-gel and dip-coating methods, atomic layer deposition is promising for ceramic membrane modification in terms of the control of layer thickness, and pore size tuning. Enhanced surface hydrophilicity and surface charge are two of the most used strategies to improve the performance of ceramic membranes for oily wastewater treatment. Nano-sized metal oxides such as TiO_2_, ZrO_2_ and Fe_2_O_3_ and graphene oxide are considered to be the potential candidates for ceramic membrane modification for flux enhancement and fouling alleviation. The passive antifouling ceramic membranes, e.g., photocatalytic and electrified ceramic membranes, have shown some potential in fouling control, oil rejection and flux enhancement, but have their limitations.

## 1. Introduction

Water scarcity is a worldwide problem that threatens the sustainable development of society [1,2]. The consumption of water by the industry sector is continuously rising in the last decades [3]. In the meantime, large amounts of wastewater are produced in various industrial processes, leading to the pollution of the aquatic environment and thus threatening human health [4,5]. One of the typical examples is oily wastewater, which is produced by several industries, such as food and beverage, and textile production, metal finishing, and oil and gas extraction [6,7,8,9]. The largest stream of oily wastewater in the world is produced during the oil and gas extraction process (produced water). It is estimated that the annual global volume of produced water reached 54 billion cubic meters in 2020 [10,11]. To deal with oily wastewater, various treatment technologies have been developed, including flotation, coagulation and flocculation, gravitational settling, hydrocyclone, and adsorption [12,13,14]. However, most of the above technologies are limited by the use of large volumes of chemical agents, the need for a large installation space, and a low separation efficiency for small oil droplets. It is therefore important to develop a robust, energy-efficient and low-cost technology for sustainable oily wastewater treatment [15].

Membrane separation is considered as one of the most promising methods for oily wastewater treatment, especially for oil/water (O/W) emulsion separation with an oil droplets size smaller than 20 µm [16,17]. Compared with traditional methods, membrane separation has a higher oil removal efficiency, a more compact design, and a smaller footprint. The high mechanical, chemical and thermal stability of ceramic membranes makes them particularly suitable for oily wastewater treatment [18,19,20,21,22]. It is assumed that ceramic membranes have a narrow pore size distribution, high porosity, and hydrophilicity. Therefore, lower fouling is commonly observed in ceramic membranes compared to their polymeric counterparts [23]. Membrane fouling, however, is an inevitable phenomenon during the separation process. For oily wastewater treatment, large oil droplets may accumulate on the membrane surface, causing cake layer formation. The membrane pore size can also be blocked or plugged by small droplets, leading to internal clogging. For both cases, the membrane performance deteriorates. This can be expressed by the reduction of water permeation flux, increase in transmembrane pressure (TMP), and increase in energy consumption [24]. To control membrane fouling, backpulsing/backwashing can be applied periodically to remove reversible fouling [25,26]. When the performance of the membrane decreases by 50–60%, chemical cleaning is needed to remove irreversible fouling and to restore the membrane performance [18]. However, the robustness of the membrane may decrease after frequent chemical cleaning, which has been found to be a challenge for ceramic nanofiltration (NF) membranes [27]. When membrane cleaning is not effective anymore, the membrane has to be replaced [28].

In order to solve the membrane fouling problem fundamentally, the most common strategy is to construct fouling-resistant membrane surfaces via membrane modification [29,30,31]. In addition, pre-coating of a protective layer on the membrane surface is found to be effective for fouling alleviation [31,32,33], but this is not covered in this review. Membrane modification is achieved by changing the surface physicochemical properties of the membrane to alleviate the interaction between the oil droplets and membrane surfaces [34,35]. It is assumed that surface hydrophilicity, charge, and roughness are the three main factors affecting membrane fouling [30]. A smooth surface is considered to be beneficial for fouling alleviation [36]. Membranes with a higher hydrophilic surface are supposed to have a lower propensity for adhesion to the hydrophobic oil droplets and are observed to have lower fouling [37,38]. For O/W emulsions stabilized by surfactants, the surface charge of the membranes becomes important for feeds with a low salinity [39]. The membrane surface can then be modified to enhance the electrostatic repulsion between oil droplets and membrane surface [40]. In this way, oil droplets are less prone to be adsorbed on the surface or inside the pores. More recently, ceramic membranes coupled with photocatalysts (e.g., TiO_2_) or Fenton catalysts (e.g., Fe_2_O_3_) have been developed to degrade the organic pollutants deposited on the surface via the strong radicals (e.g., ·OH) produced by the catalyst [41,42]. To alleviate membrane fouling, electrically active materials can also be incorporated into the matrix of ceramic membranes or coated on the membrane surface [43]. With the assistance of external electricity, the organic pollutants can then be degraded by electro-oxidation or oxidized by the strong oxidizing intermediates. Other new ceramic membranes such as piezoelectric ceramic membranes have also been developed to alleviate the fouling by oil droplets via the in-situ generated ultrasound to suppress the accumulation of oil droplets on the membrane surfaces [44,45].

In this review, we first present the various methods that are currently being used for ceramic membrane modification, the advantages and disadvantages of these methods are then discussed and compared. Afterwards, the current status, mechanism, and performance on the state-of-the-art anti-fouling ceramic membranes for the treatment of oily wastewater are presented. Finally, potential opportunities and challenges of using modified ceramic membranes for oily wastewater treatment are highlighted.

## 2. Modification Methods of Ceramic Membranes

Ceramic membranes are mostly composed of metal oxides and manufactured by high-temperature sintering. In principle, they are considered to be hydrophilic due to the presence of hydroxyl (–OH) groups on the membrane surface. However, the –OH groups density of the membrane is reduced after high-temperature calcination [46]. Surface modification of ceramic membranes can make the membranes less susceptible to fouling [30]. In 1898, Martin and Cherry reported the modification of ceramic Pasteur-Chamerland water filters for the first time with gelation or silicic acid [47]. Nowadays, many more ceramic membranes modification techniques exist, such as sol-gel [48], dip-coating [49], blending/doping [50], grafting [51], hydrothermal synthesis [52], chemical vapor deposition (CVD) [53], and atomic layer deposition (ALD) [54].

### 2.1. Sol-Gel

The most commonly used strategy for ceramic membrane modification is the sol-gel process, which is appropriate for making thin and porous layers with controllable porosity on a wide range of substrates [55]. This method provides membranes with relatively thin top layers, and it has widely been used for industrial ceramic membranes production [56]. There are two kinds of sol-gel techniques: polymerization of molecule units (PMU) and destabilization of colloidal solutions (DCS). The PMU process is controlled by the hydrolysis of alkoxides and polycondensation followed by ageing and drying in the ambient atmosphere. The DCS process uses peptization of inorganic salts or hydrous metal oxides with an electrolyte and then these colloidal solutions are destabilized and gelation is obtained [57,58]. The precursor sol can either be deposited on the membrane support to form a top layer (e.g., by dip-coating or spin coating) or cast into a suitable container with the desired shape to obtain the membrane [59]. To prevent the formation of defects and pinholes in membranes, the preparation should be done in a dust-free environment. In addition, partial gelation in the sol should be avoided [59]. Due to the particle aggregation at the sol stage in the DCS route, the PMU route has been considered to be more suitable for ceramic NF membrane fabrication [60]. The modification of ceramic membranes via the sol-gel method aims for narrowing membrane pore sizes and endowing a lower fouling surface. Bayat et al. [61] prepared a γ-alumina ultrafiltration (UF) membrane on an α-alumina substrate by the sol-gel technique for the separation of oil from real oily wastewater. The γ-alumina separation layer had an average pore size of 20.3 nm and exhibited a high permeate flux 112.7 L·m^−2^·h (LMH) and an oil rejection of 84% at a TMP of 5 bar. The low oil rejection was possibly due to the penetration of small oil droplets of feed at a relatively high temperature (35 °C).

### 2.2. Dip-Coating

The dip-coating technique, offering the advantages of flexibility and ease of operation, is also frequently used for ceramic membrane modification [49]. Dip-coating can be used for the coatings of sols or suspensions of submicrometer powders [62]. A dry substrate is dipped into a ceramic powder suspension or sol and then withdrawn from it, enabling the membrane surface to absorb a layer of suspension or sol due to the capillary forces. Once the layer comes into contact with the atmosphere it will rapidly dry and then a controlled calcination process follows [63]. Generally, the coating thickness by a dip-coating process is in the range 100 nm–100 µm [62]. Yang et al. [64] fabricated an asymmetric ZrO_2_/α-Al_2_O_3_ composite membrane with zirconia fine powder suspensions at concentrations of 5–20%. The zirconia top layer had a thickness of 20 µm and an average pore size of 0.2 µm. The performance of the prepared ZrO_2_/α-Al_2_O_3_ composite membrane was compared with three commercial alumina membranes for separating O/W emulsions and the results indicated that the zirconia membrane had the highest stable permeate flux and same oil rejection as γ-Al_2_O_3_ membrane despite its higher mean pore size.

### 2.3. Surface Grafting 

Surface grafting is achieved by the combination of polymer chains onto a solid surface via a chemical reaction process, forming a strong covalent bond between the polymer brushes and the surface [29,65]. Therefore, the grafting layer has long-term chemical stability. The precursors for grafting are mostly polymers such as fluoroalkylsilanes (FAS) [66], poly(vinylpyrrolidone) (PVP) [67], polyethylene oxide (PEO) [68] and poly(vinylacetate) (PVAc) [69]. To initialize the grafting, membrane surfaces may be pretreated by chemicals, UV-irradiation, plasma, or enzymes [70]. Depending on the kind of grafting polymers, the specific properties of the membrane can be changed from superhydrophilic to superhydrophobic, which would widen the possible application ranges of the membranes. Hydrophilic ceramic membranes, with high mechanical strength, and a high chemical and oxidant tolerance, are modified by surface grafting mainly to make them hydrophobic or superhydrophobic for membrane distillation applications [71]. However, it can also be used for ceramic membrane modification to improve its anti-fouling abilities for oily wastewater treatment. A novel fouling-resistant zirconia-based UF membrane was modified by Faibish and Cohen [65] via free-radical graft polymerization of PVP chains onto the membrane surface. The membrane was evaluated for the filtration of microemulsions with oil droplets size of 18–66 nm. Due to the effective decrease in membrane pore size by grafted PVP chains, the modified membrane demonstrated higher oil rejection compared to the pristine ceramic membrane. In addition, the modified membrane exhibited lower irreversible fouling as a result of the effective masking of –OH surface groups by PVP chains to reduce the association of solution species with the membrane surface.

### 2.4. Blending or Doping

Another membrane modification method is the blending or doping of inorganic nano-sized particles into the membrane matrix [50,72]. In this method, the nanoparticles are physically mixed with membrane precursors and then sintered at optimized temperatures via solid-state reactions. This method has also widely been applied to the fabrication of composite organic/inorganic membranes, and numerous types of inorganic materials, such as titanium dioxide (TiO_2_) [73], silicon dioxide (SiO_2_) [74], zeolites [75] and carbon nanotubes [76], have been incorporated into organic polymers for the production of NF/reverse osmosis (RO) membranes. Doping/blending can also enrich the surface functionality of ceramic membranes. Liu et al. [77] doped SiO_2_ nanoparticles into the alumina matrix and increased the hydrophilicity of the membrane, resulting in a 20.5% and 6% enhancement in water flux and oil.

### 2.5. Hydrothermal Method

Hydrothermal synthesis has widely been used for inorganic materials and zeolite membrane preparation [78,79,80]. In a typical hydrothermal reaction, an aqueous mixture of precursors is heated in an autoclave at temperatures of 80–230 °C for several hours or up to days [81]. For membrane modification, the membrane support is firstly immersed in the solution with a mixture of precursors, then the autoclave is placed in an oven for the hydrothermal process. Afterwards, the membranes are washed, dried, and calcined. This method offers several advantages such as low cost, a simple set-up, and high yield [82]. Therefore, several researchers have modified ceramic membranes via the hydrothermal process to improve their oil separation efficiency. Suresh et al. [52] prepared TiO_2_ and γ-Al_2_O_3_ composite membranes via a hydrothermal method, and due to the enhanced surface hydrophilicity, both membranes showed a higher permeate flux, while maintaining similar oil rejection for oil emulsion filtration. Paimen et al. [83] also deposited α-Fe_2_O_3_ on the Al_2_O_3_ hollow fibre support via the hydrothermal route. The deposited α-Fe_2_O_3_ layer improved the permeate flux of the membrane due to the increase in surface hydrophilicity. To remove dissolved organics in produced water, Liu et al. [84] prepared α-Al_2_O_3_ supported RO zeolite membranes by hydrothermal synthesis. The uniform sub-nanometer- or nanometer-scale pores of zeolite favoured the passage of water over organic pollutants. Therefore, the membrane obtained a high organic rejection but gave low permeance.

### 2.6. Chemical Vapour Deposition

With the CVD method the pore structure and pore size are optimized to improve the selectivity of ceramic membranes. Via the reaction of one or several gas phase precursors inside or around the substrate pores, a thin film is deposited on the porous substrate at a temperature between 400 and 1000 °C [85]. The CVD method is easier to use than the traditional sol-gel and dip-coating methods, because it does not need a repeated coating process [86]. CVD is a scalable technology, when empirical conditions for preparing good quality membranes (e.g., silica membranes) have been identified [87]. Currently, most studies about ceramic membranes modification by the CVD process have focused on the tailoring of membrane pore size for applications in gas separation [88], fuel cells [89], and catalyst membrane reactors [90]. However, the application of CVD to fabricate high-performance ceramic membranes for water treatment, especially for O/W separation, has also been reported in the literature: silica [91], silicon carbide (SiC) [40] and carbon nanotubes [92] have been studied as a coating layer for ceramic membrane modification by means of CVD to increase oil rejection and/or improve membrane fouling resistance.

### 2.7. Atomic Layer Deposition

ALD is a new technique for thin film deposition which is suitable for depositing uniform and conformal films on complex three-dimensional supports at a much lower temperature (from room temperature to 300 °C) than CVD [93]. This method was first developed in the semiconductor industry for the miniaturization of semiconductor devices [94] and then it was used for other applications such as membrane modification [95]. Compared with other deposition techniques such as CVD, molecular beam epitaxy (MBE), and evaporation, ALD can precisely control the thickness of the film at the Ångstrom or monolayer level with a high quality (pin-hole free films) [96]. With this technique, two or more precursors are made to react with one another cyclically. Normally, one reaction cycle involves four different steps: (1) exposure of the first reactant A, (2) purging of the reaction chamber to remove unreacted precursors and by-products, (3) exposure of the second reactant B, and (4) further purging to remove unreacted precursors and by-products. Depending on the required film thickness, the reaction can be repeated on the substrate surface, based on the growth cycle.

Li et al. [54] modified ceramic membranes with Al_2_O_3_ layers by the ALD deposition route. The membrane pore size decreased with the increasing ALD cycles and thus the pure water flux suffered from a decline. However, the retention to bovine serum albumin increased from 2.9% to 97.1%. Shang et al. [97] prepared a tight ceramic NF membrane by depositing a TiO_2_ layer via the ALD technique. After modification, the membrane maintained higher water permeance than commercial, tight polymer NF membranes and sol-gel-made tight ceramic NF membranes. Tight ceramic NF membranes are promising in O/W separation, especially for produced water, as they can remove micro-oil droplets, dissolved organics, and multivalent ions at the same time. However, until now ALD has only been used for modification of polymeric membranes [98] and stainless steel meshes [99] to improve their oil separation performance. To date, studies about ceramic membranes modified by ALD for O/W separation have not been carried out yet.

### 2.8. Comparison of the Different Ceramic Membrane Modification Methods

An overall comparison of the different ceramic membrane modification methods is presented in Table 1. All these modification methods have been found to be effective to improve ceramic membrane performance for oily wastewater treatment. However, these methods have their advantages and disadvantages in various aspects. Firstly, the layer thickness should be an important criterion to evaluate a modification method considering the trade-off between the membrane permeance and selectivity [100]. Sol-gel coating gives a thinner layer (50 nm–4 µm) than the dip-coating method, and thus it can be used for the preparation of ceramic NF membranes. Surface grafting aims to form polymeric chains on the membrane surface, and the layer thickness depends on the chain length of the used polymers and grafting time. Doping/blending is a physical mixing of the membrane precursors and nanoparticles, which would only change the surface functionality of ceramic membranes with a minor effect on membrane pore sizes. Zeolite RO membranes prepared by the hydrothermal method generally have a thick top layer, enabling a high rejection to organic molecules but giving low permeance [84]. Compared with the traditional ceramic membrane modification methods, CVD and ALD can obtain a relatively thin film on substrates. Especially, the ALD can achieve a thin layer with atomic layer thickness, with the potential to control membrane pore sizes at the nanoscale.

Secondly, the antifouling ability of ceramic membranes depends on the physicochemical properties (e.g., hydrophilicity and iso-electric point (IEP)) of the coated materials. In this regard, the materials that can be used for modification is another criterion when selecting the methods. Sol-gel is used for the coating of some common metal oxides such as γ-Al_2_O_3_, ZrO_2_, TiO_2_, or their mixtures [60]. However, dip-coating can be extended for almost all types of inorganic materials due to its high flexibility. Surface grafting is mainly used for the silanation of ceramic membranes to create a hydrophobic surface for membrane distillation. Doping/blending is also suitable for most inorganic materials if the doping materials can withstand a high sintering temperature as the membrane while maintaining their physicochemical properties. Hydrothermal synthesis is mainly used for the preparation of zeolite membranes. Metal oxides can also be synthesized on the membrane surface by this method, but is rarely described in literature. CVD has widely been used for the deposition of inorganic and organic thin films. For ceramic membrane modification, SiO_2_, SiC, and CNT are used for O/W separation. Similar to CVD, ALD is also used for the deposition of organic and inorganic layers on the membrane support. Therefore, CVD and ALD are supposed to be more effective than traditional sol-gel and dip-coating methods for modifications of ceramic membranes.

Lastly, the scalability and overall costs should be considered. Sol-gel and dip-coating are mature techniques for the fabrication of commercial ceramic membranes. However, defects can be formed in the selective layer, which may affect membrane performance [101]. In addition, doping/blending is a versatile and cost-effective way to modify ceramic membranes in just one step, which can save the preparation costs of the membrane while enabling the surface functionality, but is only suitable for microfiltration (MF)/UF. The long synthesis time and low permeance of the resulting membranes prepared by hydrothermal methods are problematic and, therefore, this method does not seem appropriate for membrane modification for oily wastewater treatment. CVD has been scaled up for ceramic membrane preparations mainly for gas separation [85]. The number of ceramic membranes prepared via the CVD for the water treatment field is still low possibly due to its high cost, compared with the traditional sol-gel and dip-coating methods. ALD is considered as one of the most promising methods to fine tune membrane surface properties and pore structures. However, the upscaling and the high costs of ALD is currently limiting its wide applications in the membrane field [102], but the continuous improvement and development of ALD reactors would make it feasible in the coming future.

**Table 1:** Comparison of various ceramic membrane modification methods for oily wastewater treatment.

Modification Method	Layer Thickness	Membrane Type	Layer Material	Temperature (°C)	Advantages	Disadvantages	Ref.
Sol-gel	50 nm–4 µm	UF, NF	γ-Al_2_O_3_, ZrO_2_, TiO_2,_ TiO_2_–ZrO_2_	350–600	- Narrow pore size distribution - Relatively controllable composition - Easy scale up	- Defect formation - Thick layers - Particle agglomeration	[60,61,103,104,105,106,107]
Dip-coating	100 nm–100 µm	MF, UF, NF	Most inorganic materials	800–1000	- High flexibility - Excellent homogeneity	- Susceptible to defect - Multiple coating and baking steps - Thick layers	[64,85]
Surface grafting	~	MF, UF, NF	Polymeric monomers	Room temperature	- Controllable introduction of graft chains - Simple and cheap - Long-term chemical stability	- Need initiation - Mainly for hydrophobisation of ceramic membrane	[70]
Doping/blending	~	MF, UF	Most inorganic materials	Same temperature as the membrane	- Simplicity - Cost effective - Reproducibility	- Large pore size	[50,72]
Hydrothermal synthesis	1–10 µm	MF, RO	Zeolite, TiO_2_, γ-Al_2_O_3_, Fe_2_O_3_	80–230	- Low synthesis temperature - low cost	- Limited materials - Long synthesis time - Thick layer	[81,83,84,108]
CVD	4 nm–10 µm	MF, UF, NF	Organic and inorganic materials	400–1000	- High coating uniformity - Few defects and scalable	- High deposition temperature - high cost	[85]
ALD	Monolayer to few nanometers	MF, UF, NF	Organic, inorganic and metallic materials	Room temperature to 300	- Conformal and uniform layers - Pin-hole free films - Ultrathin layers - Low deposition temperature	- Low throughput - high cost - Scale up limitation	[85,107,109]

## 3. Performance of Antifouling Ceramic Membranes for Oily Wastewater Treatment

Membrane fouling is inevitable, despite that ceramic membranes are proven to be less fouled by oil droplets compared with their polymeric counterparts [110]. Membrane characteristics including membrane pore size, surface charge, roughness, and hydrophobicity/hydrophilicity affect membrane fouling [111]. To improve the fouling resistance of ceramic membranes for oily wastewater treatment, various strategies and materials have been developed for ceramic membrane modification. Besides oil droplets, membrane fouling can also be affected by other components (e.g., surfactants, salinity, and particles) in oily wastewater [112,113]. Therefore, the modification should be tailored based on the characteristics of oily wastewater to achieve a better filtration performance. Foulants such as surfactants in oily wastewater were found to improve the flux of membranes [114], but were not part of this work. In this section, the modified ceramic membranes studied for oily wastewater treatment are discussed and compared.

Here, the ceramic membranes are further classified into active and passive antifouling membranes based on the antifouling mechanism. The active antifouling ceramic membranes mean that the fouling can be reduced or mitigated by coating a hydrophilic and/or charged layer on membrane surfaces, while the passive antifouling ceramic membranes normally need the assistance of chemicals (e.g., H_2_O_2_) or external energy (e.g., electricity, UV) to achieve their antifouling ability. Therefore, these two types of antifouling ceramic membranes are discussed separately.

### 3.1. Active Antifouling Ceramic Membranes

#### 3.1.1. Hydrophilic Ceramic Membrane

Due to the formation of a water molecule layer on the surface to prevent the contact with pollutants from the solution, a hydrophilic surface (water contact angle < 90°) is considered to be less prone to be fouled by organic compounds and microorganisms. As a result, improving surface hydrophilicity of ceramic membranes is one of the main routes to alleviate membrane fouling due to the increased affinity of the surface for water over oil [115]. Nano-scale TiO_2_, ZrO_2_, Fe_2_O_3_, γ-Al_2_O_3_, and SiO_2_ have been used to enhance the hydrophilicity of commercial ceramic membranes. Zhou et al. [37] used nano-sized ZrO_2_ to modify commercial Al_2_O_3_ membranes. The result showed that the steady flux kept 88% of the initial flux and oil rejection was higher than 97.8%, because the coating made the membrane more hydrophilic with a water contact angle of 20° on the ZrO_2_ coating. Chang et al. [38] also developed an Al_2_O_3_ supported TiO_2_ membrane with a water contact angle of 8°, which was much lower than that of the pristine membrane. The results indicated that the ceramic membrane modified by a mixture of 2 mol/L Ti(SO_4_)_2_ and 1 mol/L urea had the highest flux. In addition, TiO_2_ coating caused a higher oil rejection and lower fouling of the membrane due to a lower attraction force of the hydrophilic coating to oil droplets. In order to overcome the low flux of ceramic membranes for produced water treatment, Marzouk et al. [116] modified commercial ceramic TiO_2_ membranes with SiO_2_ nanoparticles by dip-coating. Due to the improved surface hydrophilicity, the flux and total organic carbon rejection of the membranes was improved considerably at 0.5 wt% SiO_2_ loading.

Other metal oxides such as Fe_2_O_3_, MnO_2_, CuO, and CeO_2_ have also been studied to prepare a more hydrophilic ceramic membrane. Lu et al. [117] studied the effects of the various metal oxides (i.e., TiO_2_, Fe_2_O_3_, MnO_2_, CuO, and CeO_2_) as a filtration layer on a ZrO_2_ membrane for O/W emulsion separation. Even though the surface charge of these metal oxides varies, the irreversible fouling is mainly determined by the hydrophilic character of filtration-layer metal oxides [117]. Highly hydrophilic Fe_2_O_3_ is regarded as a potential filtration-layer material for MF/UF ceramic membrane modification for O/W emulsion treatment.

Besides metal oxides, other materials, such as zeolite and activated carbon (AC), have also been used for ceramic membranes modification to improve their surface hydrophilicity. Cui et al. [108] synthesized zeolite-NaA/Al_2_O_3_ MF membranes by an in situ hydrothermal method for the separation of O/W emulsion. The pore size of NaA/Al_2_O_3_ MF membrane decreased from 2.1 µm to 1.2 µm, leading to a higher oil rejection than that of the original support. Meanwhile, the modified membrane consistently kept a higher permeate flux. This was explained by the following two reasons. Firstly, water can be transported through both inter-particle membrane pores and intra-particle zeolite pores, which enhances the permeate flux of the membranes. Secondly, the hydrophilic nature of zeolite prevents the adsorption of oil, which may result in membrane fouling. To increase the permeate flux and create lower fouling of the alumina membrane, a hybrid Al_2_O_3_/AC membrane was developed by mixing alumina powder and powdered activated carbon (PAC) sintered at 1150 °C under vacuum. The incorporation of PAC into the Al_2_O_3_ matrix created a more hydrophilic surface [118].

Due to the functional groups such as carboxyl, epoxy, and hydroxyl groups in graphene oxide (GO), a GO modified membrane is highly hydrophilic in water [119,120,121]. Hu et al. [120] prepared GO membranes on a commercial Al_2_O_3_ support and compared the performance of membranes before and after modification for filtration of O/W emulsion. The flux of the GO membrane was about 27.8% higher than that of the unmodified membrane at the same pressure and lower oil content in the permeate was observed.

#### 3.1.2. Superhydrophilic Ceramic Membrane

A superhydrophilic (water contact angle < 5°) and underwater superoleophobic (oil contact angle > 150°) membrane surface is proven to be more efficient to mitigate membrane fouling by hydrophobic oil droplets [122]. It is known that fish scales can be protected from oil contamination in water, and it was found that these surfaces have a superhydrophilic and underwater superoleophobic property [123,124]. Learning from these studies, many new materials with the same characteristics have been developed via surface engineering to broaden their applications in various fields. The hydrophilicity of a solid surface is governed by its surface free energy (chemical composition) and surface morphology (hierarchical structure) [125]. Thus, to achieve a superhydrophilic property, a surface with hierarchical macro/nanostructures and hydrophilic chemical components is necessary [126]. A membrane surface with such properties has also been developed and studied for O/W separation, where water tends to permeate through the membrane while oil is repelled [127,128,129,130].

Chen et al. [122] developed an all-inorganic ceramic membrane with superhydrophilic and underwater superoleophobic properties via dip-coating of silica nanoparticles. The results showed that the membrane displayed a higher anti-fouling ability with high oil rejection (>99.95%). In addition, the membrane was considered to have a longer service life and stronger anti-fouling ability than general polymeric membranes and traditional ceramic membranes. A superhydrophilic and underwater superoleophobic TiO_2_/Al_2_O_3_ composite membrane was designed by Zhang et al. [131], where a closely aligned TiO_2_ nanorod array was prepared on the ceramic membrane surface. Due to the superhydrophilicity and narrower pore sizes of the membrane, an ultra-low oil adhesion force (0.084 mN) was achieved, and 99.1% oils could be rejected by the membrane. Furthermore, the membrane maintained a high-water flux of 41.8 LMH under gravity.

Hydrophilic organic polymers with reactive groups are also used to prepare superhydrophilic and underwater superoleophobic ceramic membranes. Maguire-Boyle et al. [132] created a superhydrophilic surface on alumina ceramic MF membranes with cysteic acid (HO_2_CCH(NH_2_)CH_2_SO_3_H) by chemical functionalization. The TMP was much lower for the modified membrane to obtain the same flux than that of the unmodified one.

#### 3.1.3. Surface Charged Ceramic Membrane

**Figure 1:**
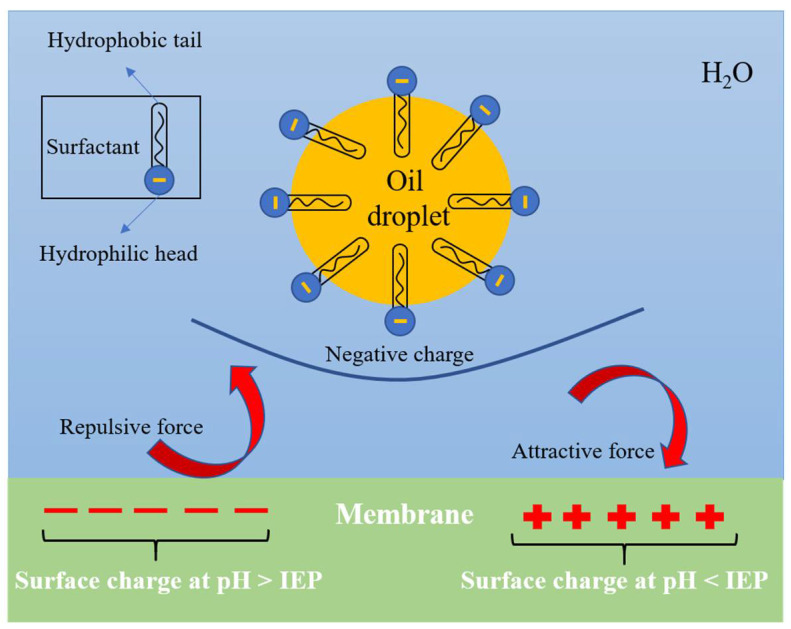
Interaction between the oil droplets stabilized by anionic surfactant and the membrane at various pH values.

Electrostatic interactions are also important for membrane rejection and fouling as it affects the interactions between solutes and membranes [39]. It has been reported that for the rejection of oil droplets, the effect of electrostatic repulsion in a membrane is more important than steric hindrance of the pore size for low ionic strength emulsions [39,133]. The surface charge of ceramic membranes is highly dependent on the pH and IEP of the ceramic materials [134]. If the pH is above the IEP of the ceramic materials, the membrane surface charge will be negative, while the membrane surface is positively charged when the pH is below the IEP [135]. Consequently, for an anionic surfactant stabilized oil emulsion, repulsive and attractive forces exist between the oil droplets and membrane surface when pH is respectively above and below IEP (schematically shown in Figure 1). The IEP of common ceramic materials in water at 25 °C are given in Table 2 below.

Most metal oxides have an IEP below 7, which means that the membranes prepared by these metal oxides have a negative charge at neutral pH. Lobo et al. [134] studied, amongst others, the influence of pH on the UF of an O/W emulsion from a metal industry. The active layer of the inorganic membrane consisted of TiO_2_ /ZrO_2_. Basic pHs enhanced membrane permeate flux, while at low pH values (pH ≤ 4), the membrane surface became positively charged and anionic surfactants adsorbed on the membrane surface, causing a flux decline and surfactant monomers permeation through the membrane.

Although the IEP of SiO_2_ is below 3, the stability of amorphous SiO_2_ in water is problematic, which limits its applications in water treatment [105,139]. Nevertheless, crystal SiO_2_, such as cristobalite and quartz, is found to be stable in water. Al-Harbi et al. [140] prepared silica-based cross-flow membranes with silica sand and glass waste as precursors for the treatment of oily wastewater. A cristobalite layer was obtained by controlled surface crystallization on the quartz surface. Although both quartz and cristobalite have a negative surface charge for pH higher than 2, the zeta potential value of the cristobalite (e.g., −42 mV at pH 6) was nearly twice lower than that of the quartz (e.g., −21 mV at pH 6) and thus a higher repulsion existed between oil droplets (negatively charged) and the membrane surface. After filtration of oily wastewater, only the filters with a cristobalite layer had a high filtration capacity with a good recovery after backwashing.

SiC and Si_3_N_4_ materials also have a very low IEP (close to SiO_2_) and high chemical stability. Xu et al. [141] compared the performance of alumina and SiC hollow fibre membranes for MF of O/W emulsion, the results indicated that the SiC membrane had a smaller pore size, but gave a higher flux due to a higher hydrophilic surface. In addition, a better anti-fouling ability was observed for the SiC membrane owing to electrostatic repulsion to oil. The application of asymmetric Si_3_N_4_ hollow fibre membrane for MF of O/W emulsion was studied by Abadikhah et al. [137]. The stable normalized permeate flux of the membrane increased from 0.2 to 0.34 when the pH of the feed emulsion increased from 2 to 12, due to the increased negative charge of Si_3_N_4_.

#### 3.1.4. Hydrophilic and Surface Charged Ceramic Membrane

Both the surface charge and hydrophilicity of the membranes can be tailored during modification, depending on the used materials. To improve the permeate flux, Zhang et al. [50] doped TiO_2_ powder to mix with Al_2_O_3_ powder to prepare a composite TiO_2_-Al_2_O_3_ ceramic membrane. The doping improved the membrane hydrophilicity and shifted the isoelectric point towards a lower pH. A higher and more stable flux was thus observed for the TiO_2_-Al_2_O_3_ composite membrane than that of the Al_2_O_3_ membrane for separation of sodium dodecyl sulfate (SDS) stabilized O/W emulsion. SiC was also doped with Al_2_O_3_ powder to prepare an Al_2_O_3_-SiC porous ceramic composite tube by extrusion. It was found that the negative surface charge and hydrophilicity of the membrane became stronger after SiC doping, and thus the SiC doped membrane demonstrated a higher permeate flux combined with a better fouling resistance [142]. More recently, Chen et al. [40] prepared a low fouling silicon carbide-alumina UF membrane for oily wastewater filtration by low-pressure chemical vapour deposition (LPCVD). The membrane had a better fouling resistance compared with the pristine alumina membrane due to a combination of improved surface hydrophilicity and negative charge. However, amphoteric bitumen in produced water can have both positive and negative surface charges. In this case, the attraction of these foulants to the ceramic membrane surface should be prevented. Atallah et al. [68] modified the γ-Al_2_O_3_ and TiO_2_ ceramic membranes with polyethylene oxide (PEO) functional organosilanes for produced water treatment. The membrane became more hydrophilic but charge-neutral. In this way, the interaction of the amphoteric bitumen in produced water with negatively charged ceramic membrane surfaces was suppressed. After modification, the membranes had a higher permeate flux as well as a higher flux recovery upon backflushing.

#### 3.1.5. Challenges of Active Antifouling Ceramic Membranes

The active antifouling ceramic membranes can slow down the membrane fouling process by minimizing the interactions between the foulants and membranes. However, an additional mass transfer resistance is usually introduced due to either an increase in the membrane top layer thickness or a decrease of membrane pore sizes [34]. The decrease of the membrane pore size can improve membrane selectivity and potentially prevent the occurrence of internal fouling, while a reduction of pure water permeance is commonly observed for the membranes after modifications [34]. To overcome this trade-off, a thin layer of highly hydrophilic materials is preferred to be used for ceramic membrane modifications to avoid the loss of water flux [143]. Then, a high mechanical and chemical stability of the coated layer should be guaranteed to extend its lifetime [143]. In addition, confined surface deposition was found to be effective to improve ceramic membrane fouling resistance with a minor effect on the water permeance [144].

Furthermore, the surface roughness is affected during modification. The increase in surface roughness can increase water flux due to a larger effective filtration area but may lead to a higher fouling potential [34]. However, regularly surface-patterned ceramic membranes, inducing turbulence of fluid on the membrane surface, are considered to increase both the flux and fouling resistance simultaneously [145]. These ceramic membranes, prepared via the 3D printing technique, may be an option for oily wastewater treatment [146].

### 3.2. Passive Antifouling Ceramic Membranes

#### 3.2.1. Photocatalytic Ceramic Membrane

**Figure 2:**
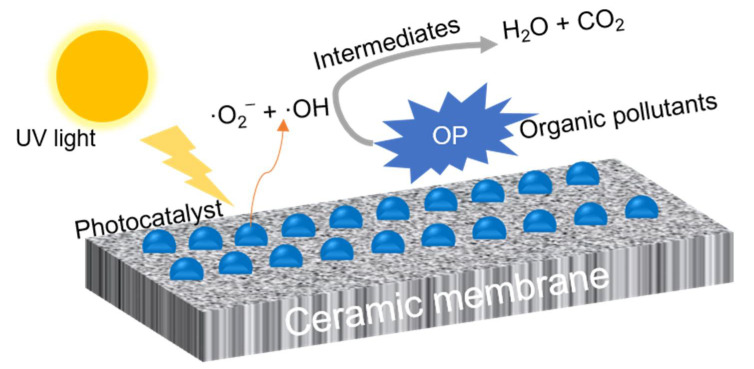
Schematic diagram depicting mechanisms that organic pollutants degradation on the photocatalytic ceramic membrane surface.

Photodegradation of organic compounds present in water and wastewaters with the application of a photocatalyst has been widely studied in literature [147]. The most suitable photocatalysts are typically made of some polycrystalline semiconductor solids such as TiO_2_, ZnO, SrTiO_3_, RuO_2_, and CdS. In particular, crystal TiO_2_ (anatase) has widely been used because of its low costs and high photostability [148]. The mechanism of the photocatalytic ceramic membrane to degrade organic pollutants can be explained with the schematic diagram shown in Figure 2. When the photocatalyst is irradiated by light (Ultraviolet (UV) or Visible), pairs of electrons and holes are excited, due to the adsorption of photons in the material. Then, the photo-generated electrons can produce active radicals such as ·O_2_^−^ and ·OH by reacting with O_2_ and H_2_O, respectively [149]. Those active species decompose organic pollutants to intermediates, CO_2_, H_2_O, and mineral salts due to the oxidizing and reducing power [150].

The performance of photocatalytic mesoporous alumina membranes was tested by Azmi et al. [151] for oil emulsion separation. The photocatalyst (copper-doped ceria) was deposited on the alumina support by a sol-gel method. Under UV irradiation, an increase in the permeance, from 36 to 1422 LMH, was observed in the separation of 1000 ppm oil emulsion. The dosage of photocatalyst on the membrane surface is quite crucial, as a too low dosage of photocatalyst leads to a lower photodegradation performance, while a high dosage may cause pore blockage of the membrane. In order to address this issue, Alias et al. [152] developed a photocatalytic nanofiber-coated (graphitic carbon nitride (g-C_3_N_4_)) ceramic membrane via electrospinning to prevent pore blockage by the photocatalysts. With the assistance of UV irradiation, a permeate flux of 640 LMH and an oil rejection of 99% were found for 180 min cross-flow oily wastewater filtration at 2 bar. Even after three cycles of 180 min filtration, the membrane could still maintain a permeate flux of 577 LMH and an oil rejection of 97%.

One of the main challenges of the photocatalytic ceramic membrane is how to illuminate the membrane surface with UV light once the membrane surface is covered with pollutants. Therefore, most studies use a flat membrane at a laboratory scale. For a single tubular membrane, the photocatalyst can be deposited on the permeate side (support layer) to be able to be illuminated by UV light [153]. For both cases, a light transmission material (e.g., quartz) needs to be used as membrane housing. In addition, the possibility of catalyst deactivation and loss during long-term filtration is problematic. The full-scale application of monolithic shaped ceramic membranes coupled with a photocatalyst is therefore still not practically feasible [154].

#### 3.2.2. Piezoelectric Ceramic Membrane

**Figure 3:**
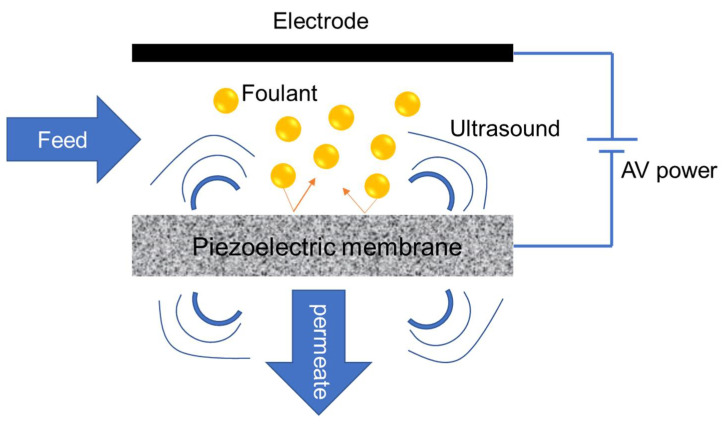
Schematic of the self-cleaning mechanism of the piezoelectric membrane. The membrane can create in-situ ultrasound to remove the pollutants deposited on the surface.

The concept of piezoelectric porous membranes for oily wastewater treatment is relatively new. These membranes can control membrane fouling by applying an alternating voltage on both sides of the membrane to generate in situ vibration from within the membrane, realizing the self-cleaning ability [45]. In addition, the investment and operation costs of this membrane are supposed to be lower as compared with the traditional membrane combined with physical and chemical cleaning [44]. A schematic diagram to show the membrane module incorporating the piezoelectric membrane and its anti-fouling performance is presented in Figure 3. The anti-fouling performance can be ascribed to vibrations and cavitation generated by the ultrasound, which prevents cake-layer formation and pollutant accumulation [155].

The first application of piezoelectric ceramic membranes for O/W emulsion separation was studied by Mao et al. [155]. Lead zirconate titanate (PZT) was used to prepare a porous ceramic membrane because of its high piezoelectric and stable porous properties. The membrane possessed the strongest vibration, with an alternating voltage signal at 190 kHz and preserved a maximum steady flux of approximately 71% of its initial value. A positive correlation with the alternate voltage amplitude and the stationary relative flux was observed for the membrane. For the studied oil concentrations (200 to 5000 ppm), the membrane with pulsed voltage always had a higher normalized stable permeance than the one without voltage applied. In addition, oil rejection by this membrane could be higher than 95%. In a following study by the same group, an Al_2_O_3_ MF membrane was prepared by dip-coating of the α-Al_2_O_3_ dispersion on the PZT ceramic substrate. The membrane had water permeance of 220 LMH/bar with an average pore size of 100 nm. With in-situ ultrasound generation during the filtration process, the stable permeate flux of the membrane improved by 48% as compared with the situation without ultrasound generation [44]. In addition, an oil rejection higher than 99.5% was observed, which was higher than that of the piezoelectric membrane in the previous study [155].

#### 3.2.3. Electrochemically Enhanced Ceramic Membrane

**Figure 4:**
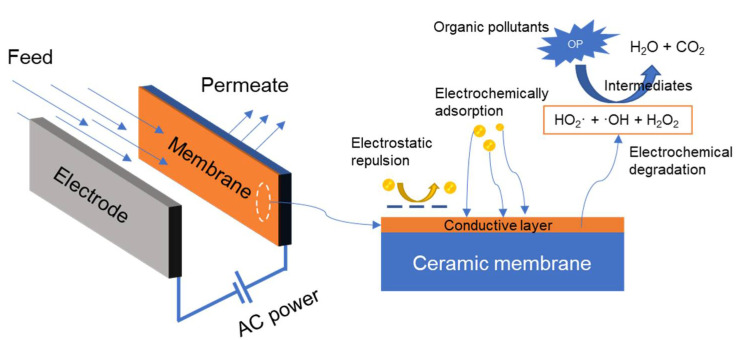
Schematic diagram of the electrochemically assisted membrane filtration process.

Electrochemically enhanced filtration is also a new technology for membrane fouling mitigation [156]. Unlike the piezoelectric membrane, the fouling mitigation of electrochemically enhanced ceramic membranes is realized via either charge inversion or electrochemical oxidation. Most foulants are charged in the wastewaters and O/W emulsion is one of the examples. To better show this filtration process, a schematic diagram is presented in Figure 4. By applying an electric field, charged particles are moved away from the membrane surface with the same charge as to the particles. In this way, the foulants such as charged oil droplets could be prevented from forming a fouling layer on the membrane surface. Alternatively, a positive charge can be applied on the conductive membrane to induce the electrosorption of negatively charged substances to improve their rejection. Afterwards, the desorption of the negatively charged substances is achieved by changing the potential periodically [157]. In addition, electrochemical reactions at the membrane surface usually occur in the electrically-assisted filtration process for electrified membranes. As a result, organic matter in the feed can be removed via the electro-oxidation process or oxidized by strong oxidizing intermediates such as ·OH, HO_2_· and H_2_O_2_ produced via directly oxidizing water molecules with the membrane as an anode. In this way, the organic fouling layer on the membrane surface or in the pores can be decomposed into intermediates, CO_2,_ and H_2_O, therefore, the membrane performance can be recovered [158].

Geng and Chen [43] studied the anti-fouling performance of tubular Al_2_O_3_ ceramic membrane after coating a layer of Magnéli Ti_4_O_7_ coupled with external electricity. The results showed that the permeate flux and oil rejection of the modified membrane were considerably improved when the electric field was applied. Moreover, the modified membranes could maintain 91.8% of their initial specific permeate flux under the applied potential of 40 V during 1 h operation. Due to the higher permeate flux and less pumping energy, the total energy consumption of electrically assisted filtration of oily wastewater decreased by 58% as compared with the pristine membrane in terms of kWh·m^−3^ permeate, even though additional DC power supply was provided. Yang et al. [159] deposited a layer of 1D IrO_2_ nanorods on an Al_2_O_3_ ceramic membrane via a dip-coating and thermal decomposition method. Due to the effect of electrochemical reactions and electrophoresis during the filtration process, the modified membrane showed a slower decline rate of permeate flux and fouling could also be minimized. In addition to flux enhancement, an increase in organic matter rejection was also achieved due to the electrochemical reactions. However, the electrophoresis effect was found to be more effective than the electrochemical effect, not only for membrane fouling control, but also for membrane cleaning.

Despite that the electrified (piezoelectric and electrochemically enhanced) ceramic membrane shows promising results in fouling control and oil rejection, this technology still faces some challenges. Firstly, the materials used to fabricate the membrane need either a high electrical conductivity or a piezoelectric property. Secondly, it is quite difficult to effectively incorporate the electrified ceramic membranes and counter electrode into a standard membrane module. In addition, at a larger system scale, to ensure a sufficiently high charge density across the membrane surface, higher potentials would be required to achieve a good filtration performance. However, this needs more corrosion resistant counter electrodes and leads to a higher energy consumption [160].

### 3.3. Comparison of the Performance of Antifouling Ceramic Membranes for Oily Wastewater Treatment

In Table 3, novel ceramic membranes and ceramic membranes modified with different materials and methods are compared for oily wastewater treatment. Because the feed characteristics and operational parameters are varied in literature, the performance of the membranes could have been influenced. Therefore, we only give a general impression on the performance of the modified ceramic membranes for oily wastewater treatment.

Considering permeate flux, oil rejection and fouling resistance, modification can effectively improve ceramic membrane performance. In terms of flux enhancement, the coating with nano-sized metal oxides (γ-Al_2_O_3_, TiO_2_, ZrO_2_ and Fe_2_O_3_) is effective in increasing the permeate flux of α-Al_2_O_3_ membranes due to the enhanced surface hydrophilicity [37,38,46]. In addition, the permeate flux of e.g., α-Al_2_O_3_ membranes could be further enhanced by GO coating. As a 2D coating material, GO has an atomic thickness with a hydrophilic property, making it a potential building block for membrane modification with a “water favoured” surface [161]. Catalytic ceramic membranes (photocatalytic and electrified membranes) have a relatively higher flux than the modified membranes with only improved surface hydrophilicity. The organic matter can be degraded by the radicals excited by the catalyst, thereby suppressing the deposition of oil droplets on the membrane surface.

Because O/W emulsion normally has a droplet size distribution in the range 1–10 µm [38,120], a high oil rejection has been found for most of the ceramic MF/UF membranes. As oil droplets are deformable, their shape and sizes can be changed depending on the operational parameters, thereby affecting the rejection [137]. The GO, Ti_4_O_7_ and g-C_3_N_4_ modified α-Al_2_O_3_ membranes are observed to have both a high oil rejection and a high permeate flux. Although the carbon nanotube (CNT) modified ceramic membrane has a very high rejection of oil droplets from the emulsion, it gives the lowest permeance, compared with the membrane modification with other materials.

TiO_2_, ZrO_2_, and Fe_2_O_3_ modified ceramic membranes have shown the lowest fouling among the nano-sized metal oxides. These metal oxides can both improve surface hydrophilicity and create a lower negative charged surface. Therefore, oil droplets are repelled from the membrane surface. GO modified ceramic membrane has a higher flux decline than metal oxides modified membranes, possibly due to its higher flux and longer filtration time. Membranes with a high initial flux are more prone to pore blocking and constriction [17]. The passive antifouling ceramic membranes, such as photocatalytic, electrified membranes, are all found to be effective for fouling alleviation. The oil droplets can be prevented from depositing on these membrane surfaces via charge inversion or degradation by radicals or in situ generated ultrasound.

## 4. Concluding Remarks

Ceramic membranes are promising for oily wastewater treatment due to their high oil removal efficiency, compact design, and a small footprint. However, membrane fouling consistently restricts process efficiency. In this review, we have compared various methods that are used for ceramic membrane modification, which have been applied for oily wastewater treatment, as well as the performance of these ceramic membranes in terms of permeate flux, oil rejection, and fouling resistance. The following conclusions can be drawn:

- Although sol-gel and dip-coating are the most frequently used methods for ceramic membranes modification, the emerging modification method ALD has shown great potential on the control of layer thickness and pore size distribution of the ceramic membranes;
- Nano-sized metal oxides have mostly been used as material for ceramic membrane modification for oily wastewater treatment. Among them, TiO_2_, ZrO_2_, and Fe_2_O_3_ are considered the most promising ones to improve ceramic membrane performance, increasing surface hydrophilicity and/or surface charge of membranes. In addition, the GO modified ceramic membranes can improve permeate flux, oil rejection, and fouling resistance at the same time. However, the scalability of these anti-fouling ceramic membranes is still challenging;
- To control the fouling problems by oil droplets, also passive antifouling ceramic membranes have been developed, including photocatalytic, piezoelectric, and electrochemically enhanced modified ceramic membranes. However, the performance of these membranes is only tested in a controlled environment for a short time, making it difficult to upscale, and the appropriate materials used to prepare these membranes are expensive.

## Figures and Tables

**Table 2:** The IEP of common ceramic materials in the water at 25 °C [135,136,137,138].

Ceramic Materials	α-Al_2_O_3_	TiO_2_	ZrO_2_	SiO_2_	SiC	Si_3_N_4_
IEP	8–9	3.9–8.2	4–6	1.8–2.7	2.6	3.3

**Table 3:** Comparison of the performance of antifouling ceramic membranes for oily wastewater treatment.

Membrane	Configuration	Method	Function	Pore Size (µm)	C_f_ (mg/L)	NF_i_ (LMH/bar)	NF_s_ (LMH/bar)	R^d^ (%)	FD (%)	FT (min)	Refs.
Fe_2_O_3_/ ZrO_2_	Disc	Pulsed-laser deposition	Hydrophilic	50 kDa	Crude oil, 100	~	~	95_COD_	12	30	[32]
γ-Al_2_O_3_/α-Al_2_O_3_	Tubular	Dip-coating	Hydrophilic/charge	0.14	Engine oil, 1000	450	315	98.5	30	60	[46]
ZrO_2_/α-Al_2_O_3_	Tubular	Dip-coating	Hydrophilic/charge	0.2	Engine oil, 1000	316	276	97.8	12.6	100	[37]
TiO_2_/ α-Al_2_O_3_	Tubular	Dip-coating	Hydrophilic/charge	0.2	Hydraulic oil, 4000	244	213	99.75	12.5	120	[38]
GO/Al_2_O_3_	Tubular	Vacuum filtration	Hydrophilic	0.2	Machine oil, 1000	950	728	98.7	23.3	140	[120]
NaA/Al_2_O_3_	Tubular	Hydrothermal synthesis	Hydrophilic	1.2	Lubricant oil, 1000	109	60	99.5	45	50	[108]
TiO_2_/Al_2_O_3_	Disc	PVD + Hydrothermal reaction	Super-hydrophilic	0.98	Toluene, 1/30 mL	287	~	93	~	~	[131]
SiO_2_/ceramic mixture	Disc	Sol-gel	Super-hydrophilic	~2	Oilfield water	~	~	99.95	~	~	[122]
TiO_2_-doped α-Al_2_O_3_	Tubular	Doping + sintering	Negative charge	0.2	Soybean oil, 5000	705	605	~	14	120	[50]
CNT/YSZ	Disc	CVD	Increase rejection	0.7	Blue oil based ink, 210	36	26	100	28.6	550	[92]
IrO_2_/Al_2_O_3_	~	Dip-coating	Electrochemical degradation	<1	Peanut oil, 200	414	331	96.3_COD_	20	160	[159]
Ti_4_O_7_/Al_2_O_3_	Tubular	Dip-coating	Electrochemical degradation	0.2	Peanut oil, 200	1018	914	97.9_COD_	9.2	60	[43]
CuO/CeO_2_/ Al_2_O_3_	Hollow fiber	Sol-gel	Photocatalytic degradation	0.05	1000	1815	1422	92	22	240	[151]
g-C_3_N_4_/Al_2_O_3_	Hollow fiber	Electro-spinning	Photocatalytic degradation	0.25	Crude oil, 1000	816	640	99_TOC_	21	180	[152]
PZT	Disc	~	Generated ultrasound	0.3	Soybean oil, 500	86	73.1	95.3_TOC_	15	180	[155]
Al_2_O_3_/ PZT	Disc	Dip-coating	Generated ultrasound	0.1	Soybean oil, 200	230	185	99.5_TOC_	20	120	[44]

C_f_—Oil concentration in the feed, NF_i_—Normalized initial flux, NF_s_—Normalized stable flux, R^d^—Oil rejection, FD—Flux decline percentage, FT—Filtration time, ~ not reported, a—Permeate volume (mL), PVD—physical vapor deposition.

## Data Availability

Not applicable.
